# Supplementary material for: Mesoscopic superconductivity and high spin polarization coexisting at metallic point contacts on Weyl semimetal TaAs
Source: Nat Commun. 2017 Jan 10;8:13974. doi: 10.1038/ncomms13974 (PMC5234076; doi:10.1038/ncomms13974)
Supplement: Supplementary Information — Supplementary Figures 1-9, Supplementary Table 1 and Supplementary Note 1 and Supplementary References [file ncomms13974-s1.pdf]

Supplementary Figures

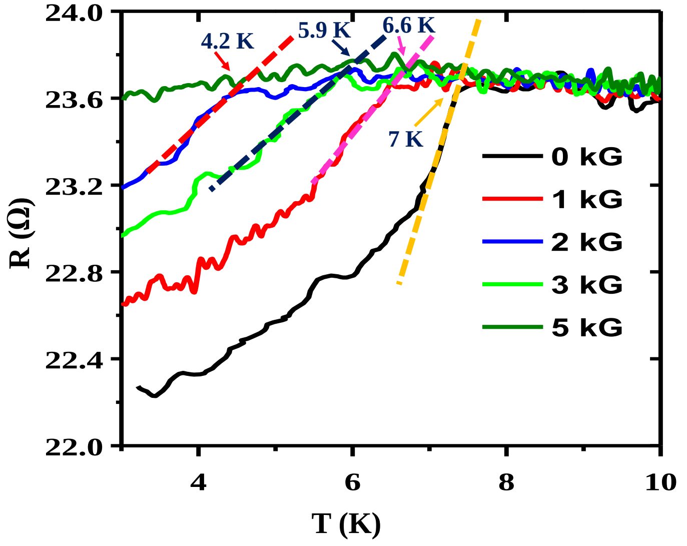

SupplementaryFigure 1: **Scheme for $T_\mathbf{c}$ determination:** The data depict the temperature dependence of point contact resistance ($R-T$) for a thermal regime point contact. The superconducting transition temperature (T_c) has been determined by drawing two slope to the $R-T$ data, one for the part above the transition and the other for the part below the transition. The point where the two lines meet has been defined as the critical temperature T_c. The magnetic field evolution of the $R-T$ data is also shown where the decrease in T_c at higher fields is clearly visible.

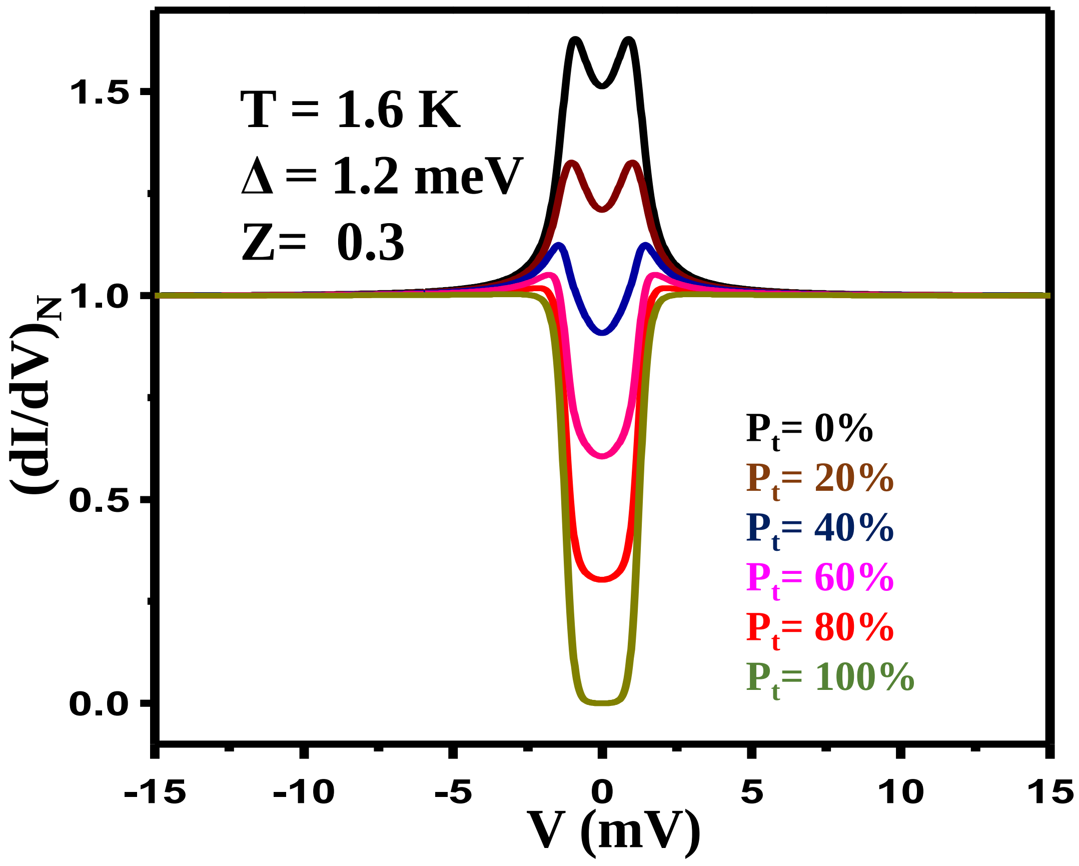

SupplementaryFigure 2: Simulated spectra for different degree of spin polarization of the transport current flowing through the point contacts using the modified BTK theory.

Single crystal X-ray diffraction

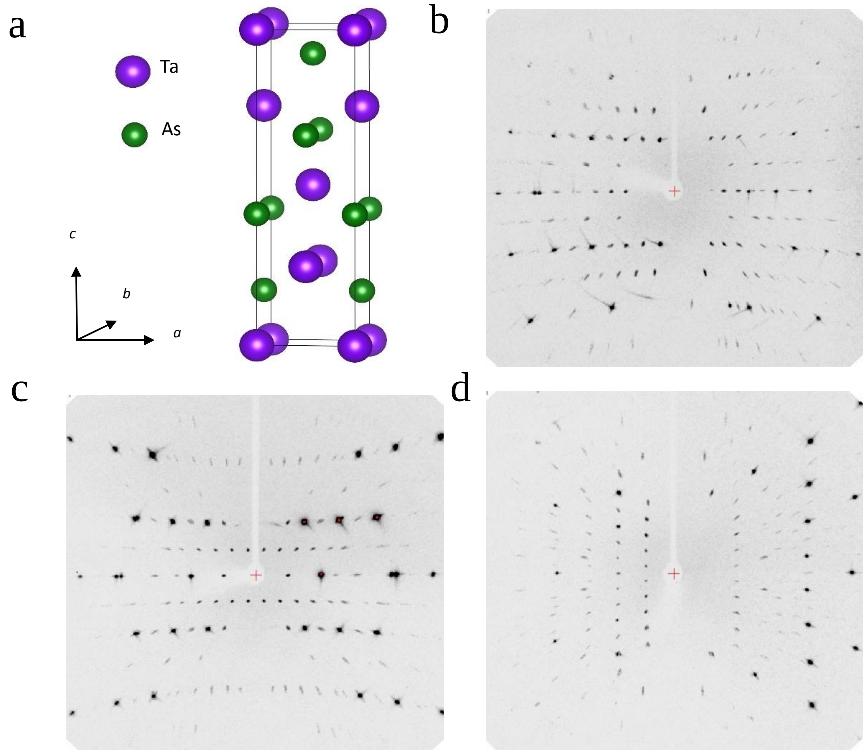

SupplementaryFigure 3: **Unit cell and x-ray diffraction of TaAs crystal:** (a) a non-centrosymmetric unit cell of TaAs. (b, (c) and (d), show the rotating x-ray diffraction patterns of the TaAs crystal about the crystallographic a, b, and c-axis respectively. For each case, the rotating axis is vertical.

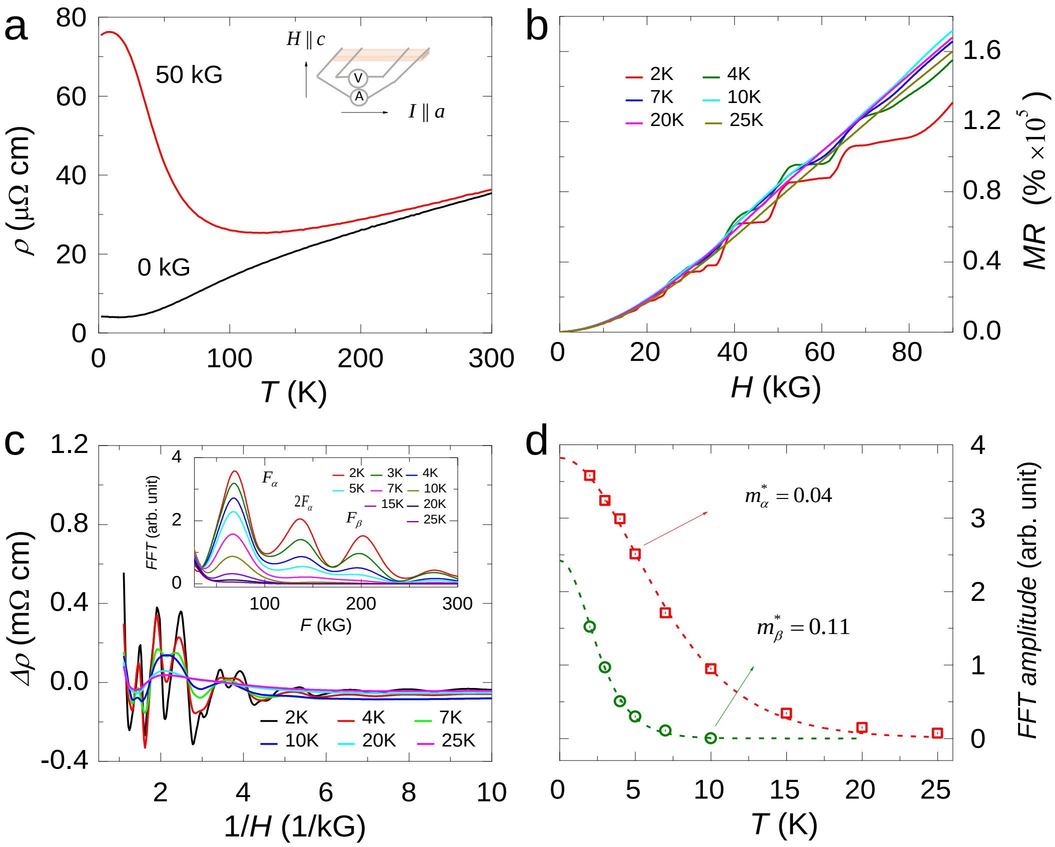

SupplementaryFigure 4: **Magneto-resistivity at various temperatures:** (a) temperature dependent resistivity, ρ (T) at 0 field and 50 kG field, (b) magnetoresistance (MR) up to 90 kG at different temperatures. A large amplitude of Shubnikov de-Haas can be seen up to 25 K, (c) (SdH) oscillations after subtracting a cubic polynomial. Fast Fourier transform (FFT) of SdH oscillations mainly gives two frequencies at F_α= 68 kG, F_β = 198 kG (inset). (d) Effective mass corresponding to frequencies F_α and F_β.

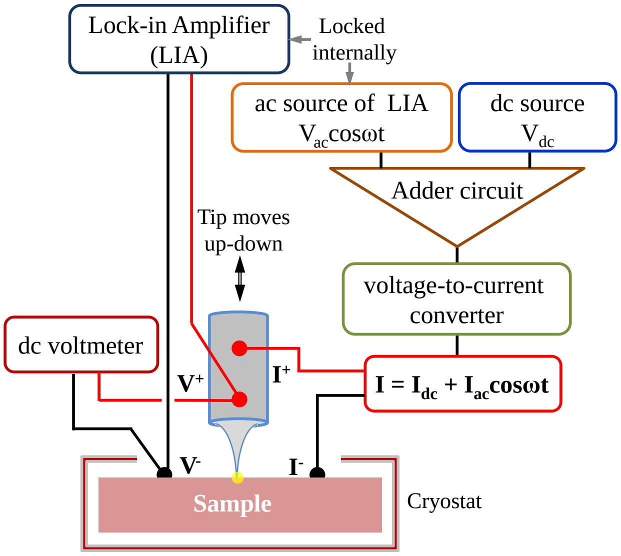

SupplementaryFigure 5: **Point-contact Spectroscopy:** Schematic diagram describing the point-contact spectroscopy measurements.

Additional data in the thermal regime

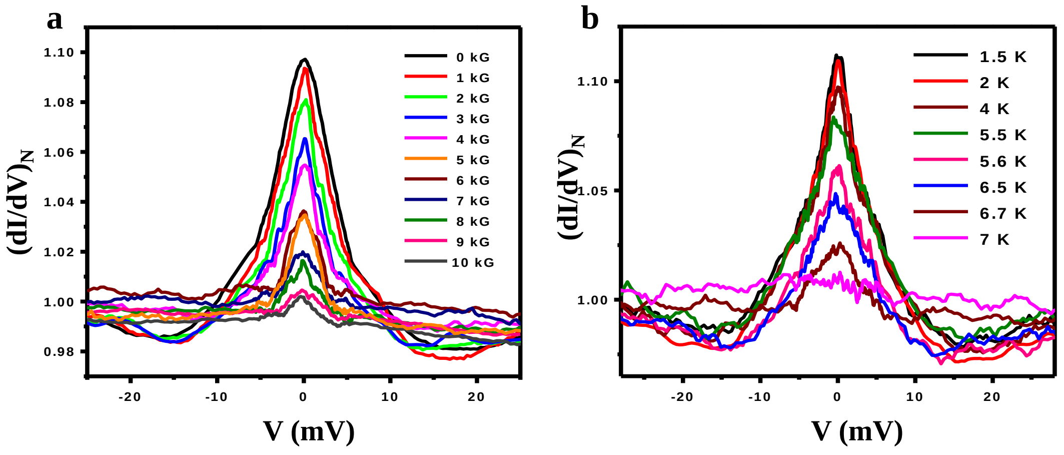

SupplementaryFigure 6: Magnetic field and temperature dependence of another point contact in the thermal regime.

Additional spectroscopic data in the ballistic regime obtained on crystal B

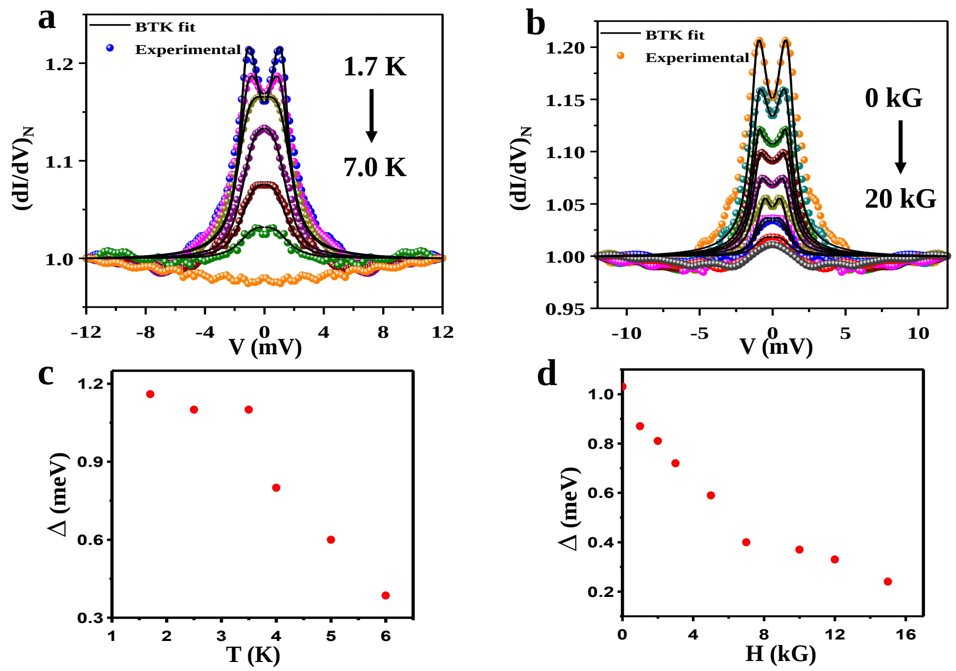

SupplementaryFigure 7: **Magnetic field and temperature dependence of another point contact in the ballistic regime obtained on crystal B:** (a) Temperature dependence of the ballistic limit spectra (coloured dots) along with modified BTK fit (solid black lines), (b) Magnetic field dependence of the ballistic limit spectra (colored dots) along with modified BTK fit (solid black lines). (c) Temperature dependence of the gap (Δ). (d) H-dependence of the gap (Δ).

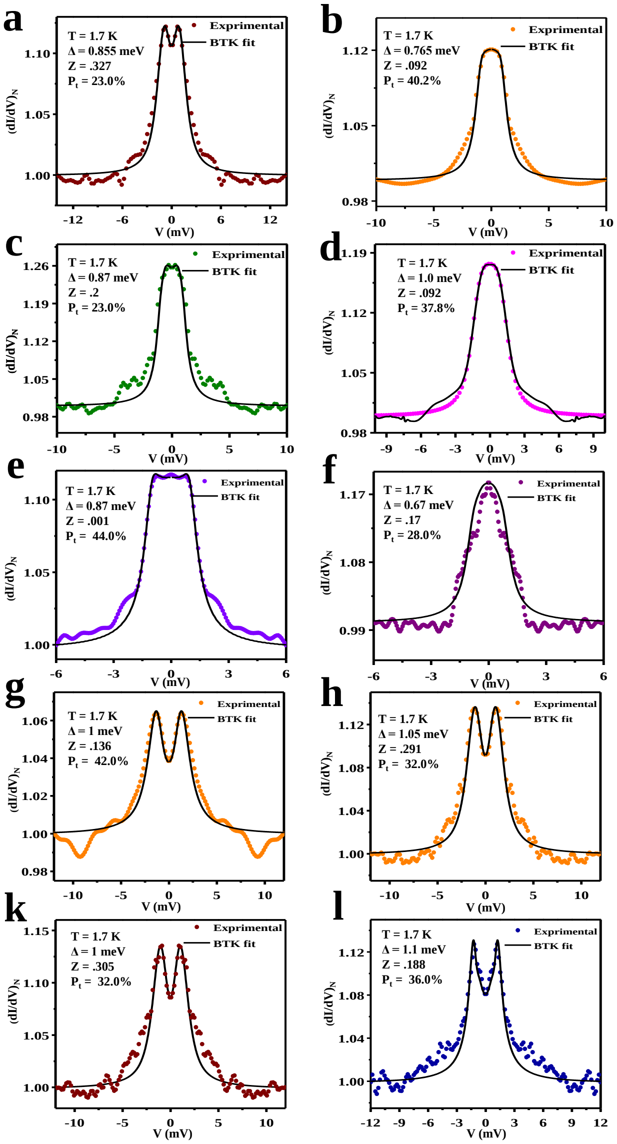

SupplementaryFigure 8: A collection of data and their modified BTK fit used for extracting spin polarization. As can be seen in the different panels of this Figure, the spin polarization ($P_{\rm t}$) varies for different Z. The intrinsic spin polarization was estimated by plotting $P_{\rm t}$ vs. Z and extrapolating the plot to $Z = 0$.

A collection of different types of contact-geometry dependent data obtained in the thermal regime

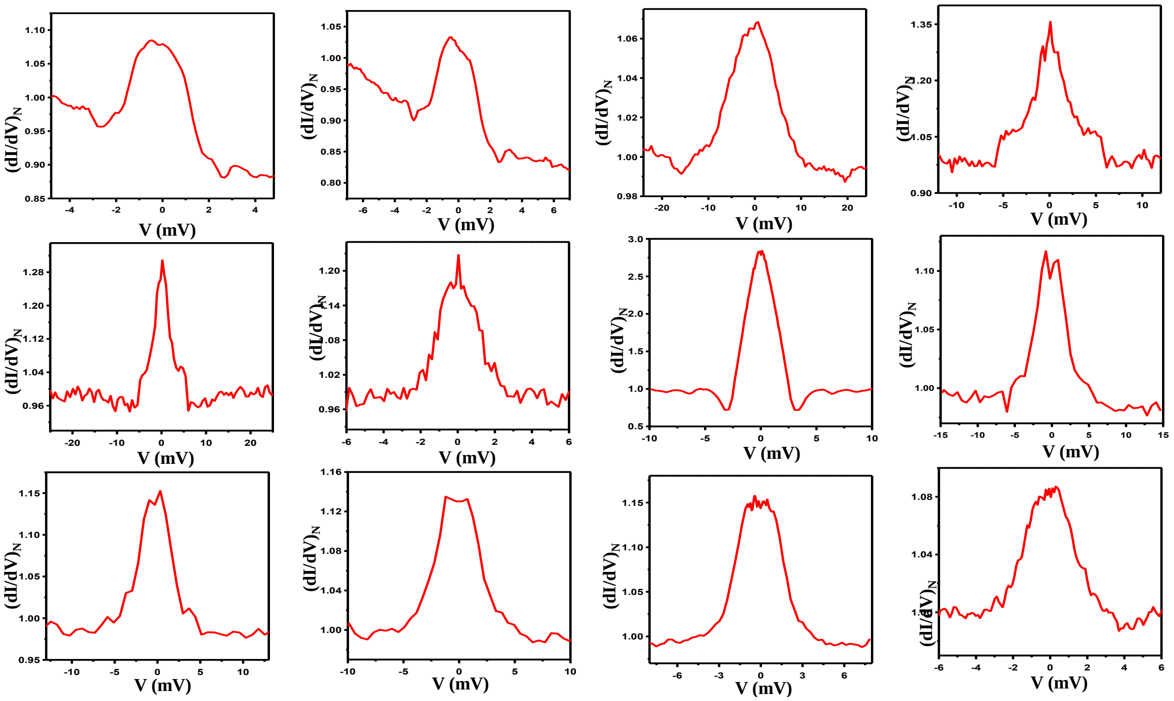

SupplementaryFigure 9: A collection of thermal limit point-contacts showing geometry dependence of the point contacts.

Supplementary Table

S. No. of Point - Contact	R_{PC} (Ω)	T (K)	Delta (meV)	P (%)	Z	Gamma (Γ)
1	0.39	1.7	1	42	0.136	0.075
2	1.64	1.7	1.1	31	0.279	0.012
3	0.072	1.7	0.8	49.5	0.08	0.006
4	0.93	1.7	1.26	34	0.242	0.001
5	3.01	1.7	0.98	33.3	0.255	0.07
6	0.66	1.7	1.1	36	0.188	0.1
7	0.485	1.7	0.87	23	0.2	0.109
8	0.71	1.7	1	37.8	0.092	0.018
9	0.23	1.7	0.87	44	0.001	0.001
10	0.274	1.7	0.67	28	0.17	0.095
11	0.71	1.7	1.05	32	0.291	0.071
12	0.7	1.7	1	32	0.305	0.06
13	0.85	1.7	0.765	40.2	0.092	0.017

Supplementary Table I: Fitting parameters used to fit certain point contacts in the ballistic/diffusive regime.

Supplementary Note 1

Different regimes of transport

We achieved different point contact transport regimes by tuning the size of point contact through mechanical movement of the tip (this is known as needle-anvil method). In our work we have used conventional definition of various regimes as discussed below:

1. Ballistic regime: The contact size is smaller than the elastic mean free path. This means, the electrons statistically do not undergo scattering and therefore, the electron does not dissipate energy within the contact region. Spectroscopic studies can be possible in this regime. Both energy and momentum resolved spectroscopy are done in this regime.

2. Diffusive regime: The contact size is larger than the elastic mean free path but still smaller than the inelastic mean free path. Elastic scattering is allowed and therefore, momentum resolved spectroscopy cannot be performed in this regime. However, this is still a spectroscopic regime and energy resolved spectroscopy can be done equally well as in a ballistic regime.

3. Thermal regime: The contact size is larger than the inelastic mean free path. No spectroscopy can be performed in this regime due to dissipation.

Now, in our paper, from the spectral features, we can conclude that our point contacts were in the ballistic or diffusive regime of transport and that is why we obtained energy resolved spectroscopic information. We have also defined another regime called the intermediate regime (the term has been borrowed from Wexler's theory[1]), where the contact size falls between the spectroscopic regime (ballistic/diffusive) and thermal regime.

How did we determine the critical temperature (T_c)?

For all the point-contacts reported here we have measured the temperature (T) dependence of the point-contact resistance (R) with $V_{DC}=0$. The $R - T$ data show a broad transition to the superconducting state. We have drawn the slope of the $R - T$ curves above and below the onset of transitions (see supplementary fig 1). The temperature at which the two slopes for a given $R - T$ curve meet has been taken as the T_c for the corresponding point-contacts. It is important to note that for the ballistic point-contacts we cannot measure the T_c as for such point-contacts the contact-resistance depends only on fundamental constants and remain temperature independent. However, from the thermal

limit point-contacts we learn that the T_c does not have a strong dependence on contact size for a given sample.

Analysis of the ballistic regime data: Modified BTK theory with spin polarization

As per BTK theory[2,3] when the point contact is between a superconductor and an unpolarized (normal) metal, the current is given by

I_u= $C\int_{-\infty}^{+\infty}[f(E-eV)-f(E)]$ [1+$A_u(E)$- $B_u(E)$] dE

where, $A_u(E)$ is the Andreev reflection probability and $B_u(E)$ is the normal reflection probability for an unpolarized current.

For a fully polarized current, the same formula can be used by replacing A_u and B_u with A_p and B_p respectively, where, A_p and B_p are respective probabilities for a fully spin polarized Fermi surface of the non-superconducting electrode forming the point contact.

The subscripts u and p denote the coefficients for the unpolarized and polarized current, respectively.

$A_u(E)$, $B_u(E)$, $A_p(E)$, $B_p(E)$ were calculated using the following formula:

$A_p(E)$=0 for the entire range of E,

$B_p(E)$=1, for $E<\Delta$,

$$B_p(E)=\frac{(\sqrt{\frac{E^2-\Delta^2}{E^2}}-1)^2+4Z^2(\frac{E^2-\Delta^2}{E^2})}{(\sqrt{\frac{E^2-\Delta^2}{E^2}}+1)^2+4Z^2(\frac{E^2-\Delta^2}{E^2})}, \text{ for } E>\Delta$$

$$A_u(E)=\frac{\Delta^2}{E^2+(\Delta^2-E^2)(1+2Z^2)^2}, \text{ for } E<\Delta,$$

$A_u(E)=\frac{(u^s v^s)^2}{\gamma^2}$, for $E>\Delta$

$B_u(E)=1-A(E)$, for $E<\Delta$,

$B_u(E)=\frac{((u^s)^2-(v^s)^2))^2Z^2(1+Z^2)}{\gamma^2}$, for $E>\Delta$

$\gamma^2=(((u^s)^2-(v^s)^2)Z^2+(u^s)^2)^2$,

Here u^s and v^s are obtained from the solution of the Bogoliubov-de Gennes (BdG) equation in the superconductor:

$$(u^s)^2=1-(v^s)^2=1/2[1+\sqrt{\frac{E^2-\Delta^2}{E^2}}]$$

$$Z=\frac{V_0}{\hbar v_F}$$

Z is the dimensionless parameter used in BTK theory and it is directly proportional to the strength of the potential barrier at the point-contact interface. The constant C has been

determined by matching the scales of the experimental data and the theoretical curves.

After calculating the I_{u} and I_{p} we have simply written the total modified current including the transport spin polarization P_{t} as

$I_{\mathrm{mod}} = (1 - P_{\mathrm{t}})I_{\mathrm{u}} + P_{\mathrm{t}}I_{\mathrm{u}}$

This is used to fit the experimental dI/dV curves to determine the transport spin polariztion P_{t}.[4]

The magnitude of spin polarization (P_{t}) thus determined may decrease monotonically with increasing barrier strength (Z). Such a dependence is seen routinely in spin polarization measurements using Andreev reflection spectroscopy and is attributed to spin-fip scattering processes taking place at mesoscopic interfaces with higher barrier strength.[5–7] For certain materials, however, where such spin-dependent scattering processes are not allowed, the dependence of P_{t} on Z may not be observed.

Several point contacts also showed small amount of broadening. Such broadening could be accounted for by adding an imaginary component Γ to Energy E, i.e., $E \rightarrow E + i\Gamma$. In such fittings, the value of Γ was constrained to be very small, i.e., smaller than 10% of Δ. Physically adding an imaginary component to the energy means introducing finite lifetime of the quasiparticles. Therefore, Γ should be small compared to Δ. Otherwise, the superconducting phase cannot be stable.

Supplementary References

[1] Wexler, A. The size effect and the non-local Boltzmann transport equation in orifice and disk geometry. *Proc. Phys. Soc.* **89**, 927-941 (1966).

[2] Blonder, G. E., Tinkham, M. & Klapwijk, T. M. Transition from metallic to tunneling regimes in superconducting microconstrictions: Excess current,charge imbalance, and supercurrent conversion. *Phys. Rev. B* **25**, 4515 (1982).

[3] Soulen, R. J. *et al.* Measuring the spin polarization of a metal with a superconducting point contact. *Science* **282**, 85 (1998).

[4] Mazin, I. I. How to define and calculate the degree of spin polarization in ferromagnets. *Phys. Rev. Lett.* **83**, 1427 (1999).

[5] Anshu, S. *et al.* High spin polazation and the origin of a unique ferromagnetic ground state $CuFeSb$. *Appl. Phys. Lett.*, **108** 242411 (2016).

[6] Raychaudhuri, P., Mackenzie, A. P., Reiner, J. W. & Beasley, M.R. Transport spin polarization in $SrRuO_3$ measured through point-contact Andreev reflection. *Phys. Rev. B* **67**, 020411 (2003).

[7] Woods G. T. *et al.* Analysis of point-contact Andreev reflection spectra in spin polarization measurements. *Phys. Rev. B* **70**, 054416 (2004).
